# Reverse-engineering method for XPCS studies of non-equilibrium dynamics

**DOI:** 10.1107/S2052252522004560

**Published:** 2022-05-28

**Authors:** Anastasia Ragulskaya, Vladimir Starostin, Nafisa Begam, Anita Girelli, Hendrik Rahmann, Mario Reiser, Fabian Westermeier, Michael Sprung, Fajun Zhang, Christian Gutt, Frank Schreiber

**Affiliations:** a Institute of Applied Physics, University of Tübingen, Auf der Morgenstelle 10, 72076 Tübingen, Germany; b Department of Physics, University of Siegen, Emmy-Noether-Campus, Walter-Flex-Straße 3, 57076 Siegen, Germany; c European X-ray free-electron laser GmbH, Holzkoppel 4, 22869 Schenefeld, Germany; d Deutsches Elektronen-Synchrotron DESY, Notkestraße 85, D-22607 Hamburg, Germany

**Keywords:** small-angle X-ray spectroscopy, dynamical simulations, phase transitions, X-ray photon correlation spectroscopy, liquid–liquid phase separation, proteins, reverse engineering, non-equilibrium dynamics

## Abstract

X-ray photon correlation spectroscopy (XPCS) is a powerful tool in the investigation of dynamics covering a broad time and length scale. It has been widely used to probe dynamics for systems in both equilibrium and non-equilibrium states; in particular, for systems undergoing a phase transition where the structural growth kinetics and the microscopic dynamics are strongly intertwined. The resulting time-dependent dynamic behavior can be described using the two-time correlation function (TTC), which, however, often contains more interesting features than the component along the diagonal, and cannot be easily interpreted via the classical simulation methods. Here, a reverse engineering (RE) approach is proposed based on particle-based heuristic simulations. This approach is applied to an XPCS measurement on a protein solution undergoing a liquid–liquid phase separation. It is demonstrated that the rich features of experimental TTCs can be well connected with the key control parameters including size distribution, concentration, viscosity and mobility of domains. The dynamic information obtained from this RE analysis goes beyond the existing theory. The RE approach established in this work is applicable for other processes such as film growth, coarsening or evolving systems.

## Abstract 2

A novel reverse engineering (RE) approach is presented based on particle-based heuristic simulations for the understanding of dynamics in non-equilibrium systems revealed by X-ray photon correlation spectroscopy measurements. The RE approach provides a direct connection between the experimental dynamic features and the key control parameters of the non-equilibrium process. This framework is also applicable to other related processes.

## 1. Introduction

Simulations can be considered the third pillar alongside theory and experiment in modern physics (Riedel *et al.*, 2009 ▸; Sabelli, 2006 ▸), and simulations based on theoretical equations are often used to compare and rationalize the experimental results. However, this approach has limitations related to the assumptions used to derive the theory. In particular, when more complex phenomena are involved, for which a comprehensive theory is not available. In view of this, a different approach is needed that focuses on the relation between the phenomenon and the key control parameters instead of a specific theory.

For example, understanding the dynamics of proteins in solutions is extremely important for their complex phase transitions including liquid–liquid phase separation (LLPS) (Wang *et al.*, 2013 ▸), protein crystallization (Durbin & Feher, 1996 ▸), glass transition (Cardinaux *et al.*, 2007 ▸), bio-materialization (Gunton *et al.*, 2007 ▸), and in the food and pharmaceutical industry. The dynamic behavior of these non-equilibrium process covers length scales ranging from single-protein size to micrometre phase domains and time scales from microseconds to hundreds of seconds (Da Vela *et al.*, 2016 ▸; Beck *et al.*, 2019 ▸; Banc *et al.*, 2019 ▸). To date, a theory describing these dynamics does not exist.

Experimentally, high-brilliance third- and fourth-generation synchrotron light sources have made it possible to carry out X-ray photon correlation spectroscopy studies (XPCS) to cover the broad range of dynamics. Nowadays, XPCS is widely used in different areas of soft condensed matter research, *e.g.* for studying the dynamics of colloids (Lurio *et al.*, 2000 ▸), liquids (Seydel *et al.*, 2001 ▸), polymers (Lumma *et al.*, 2001 ▸) and clays (Bandyopadhyay *et al.*, 2004 ▸). Recent progress in the experimental and conceptual frameworks has allowed the difficulties in working with beam-sensitive samples (Jeffries *et al.*, 2015 ▸; Fluerasu *et al.*, 2008 ▸; Ruta *et al.*, 2017 ▸; Verwohlt *et al.*, 2018 ▸) and often called bio-XPCS (Möller *et al.*, 2019 ▸; Vodnala *et al.*, 2016 ▸; Ragulskaya *et al.*, 2021 ▸; Girelli *et al.*, 2021 ▸; Begam *et al.*, 2021 ▸; Lurio *et al.*, 2021 ▸) to be overcome.

Time-dependent dynamics of XPCS studies for non-equilibrium processes are frequently presented using two-time correlation functions (TTCs) (Headrick *et al.*, 2019 ▸; Fluerasu *et al.*, 2007 ▸; Ehrburger-Dolle *et al.*, 2019 ▸; van t Zand *et al.*, 2012 ▸; Conrad *et al.*, 2015 ▸; Hernández *et al.*, 2014 ▸; Ju *et al.*, 2019 ▸; Madsen *et al.*, 2010 ▸). The rich features of TTCs are closely related to non-equilibrium dynamics; however, interpretation of TTCs often focuses only on the component along the diagonal of TTCs (Fluerasu *et al.*, 2007 ▸; Ehrburger-Dolle *et al.*, 2019 ▸; van t Zand *et al.*, 2012 ▸; Conrad *et al.*, 2015 ▸; Hernández *et al.*, 2014 ▸), and further extraction of their relaxation time and Kohlrausch exponent for the description of the investigated dynamics (Sinha *et al.*, 2014 ▸). A recent study of thin-film growth on polycrystalline surfaces (Headrick *et al.*, 2019 ▸) has demonstrated that the observed ‘side’ features in the TTC are related to the step-edge velocity. An XPCS study of layer-by-layer crystal growth (Ju *et al.*, 2019 ▸) shows that additional features in the TTC are connected to the memory effect in the arrangement of islands formed on successive crystal layers. In both studies, equation-based numerical modeling was used to rationalize the experimental results. Such a simulation of complex multi-component processes may confirm the existence of certain features but cannot unambiguously pinpoint their physical origin. In this case, another simulation approach, *i.e.* controlled variation of the key parameters in the equations to study the dependence of TTC features on these parameters, but not directly their dependence on the dynamical phenomena. For a more detailed investigation of the connection between the dynamics and the obtained TTC features, it is desirable to decouple the complex dynamics of the system into sub-phenomena. This approach can be realized via simulations based on the reverse engineering (RE) technique (Eilam, 2005 ▸; Buonamici *et al.*, 2018 ▸; Asadizanjani *et al.*, 2017 ▸). In essence, RE is the deconvolution of a complex system into simple components followed by the analysis of how these components work independently and how they interact. The RE approach has been applied in different research areas, such as design of soft materials with the desired properties and functions (Dijkstra & Luijten, 2021 ▸; Sherman *et al.*, 2020 ▸; Ferguson, 2017 ▸).

In this work, we propose an RE approach based on particle-based heuristic simulations to understand the complex dynamic features of TTCs obtained from XPCS measurements. The system studied is bovine serum albumin (BSA) solution in the presence of trivalent salt YCl_3_ (Matsarskaia *et al.*, 2016 ▸). The kinetics of LLPS have been studied previously and a tendency to a kinetically arrested state is observed (Da Vela *et al.*, 2016 ▸, 2020 ▸), while non-equilibrium dynamics have been studied recently using XPCS (Ragulskaya *et al.*, 2021 ▸). This work is a step forward for the investigation of the dynamics of LLPS (Alberti *et al.*, 2019 ▸; Alberti & Dormann, 2019 ▸; Hyman *et al.*, 2014 ▸; Ragulskaya *et al.*, 2021 ▸; Girelli *et al.*, 2021 ▸) on the micrometre scale of phase-separating domains. The main objective of this work is to advance the field of XPCS analysis and improve the knowledge on the features displayed in TTCs, their possible explanation and the connection between them, and the key control parameters of the non-equilibrium dynamics. The RE approach may be successful in going beyond the existing theory if there is no theoretical formalism for the investigated phenomenon or if the experiment cannot be fully described by theory.

## 2. Experimental methods

BSA and YCl_3_ were purchased from Sigma–Aldrich. Samples were prepared following previous work (Da Vela *et al.*, 2016 ▸, 2020 ▸). The stock solutions of protein and salt were mixed at 21°C with an initial protein concentration of 175 mg ml^−1^ and a salt concentration of 42 m*M*. The obtained solution was stabilized and centrifuged, and the dense phase was used for further experiments.

XPCS experiments were performed in an ultra small angle X-ray spectroscopy (USAXS) geometry at the PETRA III beamline P10 (DESY, Hamburg, Germany) at an incident X-ray energy of 8.54 keV (λ = 1.452 Å) and a beam size of 100 × 100 µm^2^. The sample-to-detector distance was 21.2 m, which corresponds to a *q* range of 3.2 × 10^−3^ nm^−1^ to 3.35 × 10^−2^ nm^−1^, where 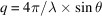 and 2θ is the scattering angle. The data were collected by an EIGER X 4M detector from Dectris with 75 × 75 µm^2^ pixel size. The sample was filled into capillaries of 1.5 mm in diameter. The phase transition from one- to two-phase regime was started by a rapid temperature jump (Ragulskaya *et al.*, 2021 ▸; Da Vela *et al.*, 2020 ▸). Measurement details can be found in the supporting information.

During XPCS measurements, a series of 2D speckle patterns was collected, and the TTC *G*(*q*, *t*_1_, *t*_2_) can be calculated (Grübel *et al.*, 2008 ▸) for a fixed *q* as

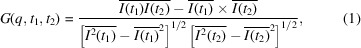

where the average is performed over pixels within the same momentum transfer *q* ± Δ*q*, and *t*_1_ and *t*_2_ are the times at which the intensity correlation is calculated.

## 3. Results and discussion

### 3.1. Dynamic map from XPCS measurements

**Figure 1:**
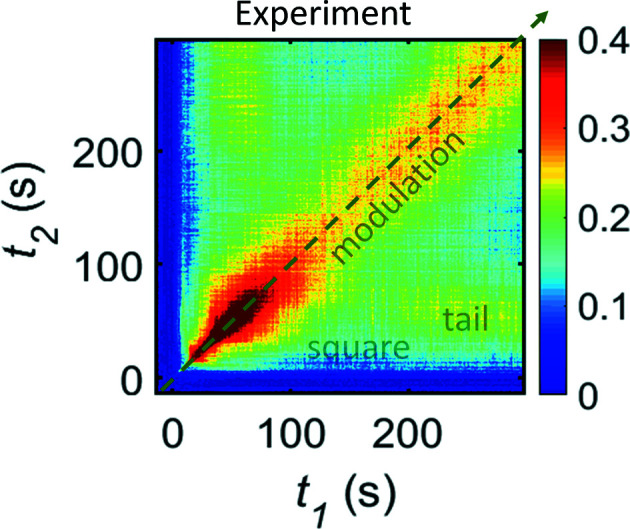
Experimental TTC for the BSA—YCl_3_ sample undergoing LLPS. XPCS measurement was performed for a temperature jump from 10 to 40°C with a total measuring time of 312 s. The TTC is for *q* = 4.4 µm^−1^. The square, tail and modulation features are marked with half-transparent text. The green dashed line shows a direction of increase of experimental time *t*_age_.

A typical experimental TTC for LLPS on a micrometre-length scale is presented in Fig. 1 ▸. A relaxation signal appears a few seconds after the temperature jump, initially with a fast decay rate, but quickly broadening. After the quick broadening, the main relaxation along the diagonal turns into a steady state. A new slow relaxation mode gradually appears, and its correlation shows a decay around 20–30 s, leading to a ‘square’ feature in the TTC.

The typical TTC discussed here contains three characteristic features: ‘modulation’ along the diagonal, a square feature, and a ‘tail’ (follow labels in Fig. S1 of the supporting information). The modulation represents the main component of the dynamics. The square feature is presented as the frozen-in component that is visible as a square-like background (Fig. S2). Tails are oscillations of the *G*(*q*, *t*_1_, *t*_2_) values along the *t*_1_ axis. In Fig. 1 ▸ these tails are areas of high contrast around *t*_1_ ≃ 230 s and *t*_2_ ≃ 50 s and are symmetrical to the diagonal one. Both, square and tail features, exhibit strong *q* dependency. They move to earlier times *t*_age_ with increasing *q*, which means that processes corresponding to these features are size sensitive and detectable earlier for smaller sizes on the microscopic length scale [follow Figs. S2(*a*)–S2(*d*)]. Another important feature of experimental TTCs is the fluctuation of the contrast near the diagonal with time *t*_age_: initially there is a rapid increase followed by a moderate gradual decline. Classical analysis of the TTC for LLPS systems with the extraction of the relaxation times and Kohlrausch exponents evolution can be obtained from Ragulskaya *et al.* (2021 ▸) and Girelli *et al.* (2021 ▸). Here, we focus on the relation between the observed TTC features and the control parameters of the dynamic process.

### 3.2. Classical Cahn–Hilliard simulations and binarization

The evolution of the spinodal decomposition in real space can be described by the Cahn–Hilliard (CH) equation (Cahn & Hilliard, 1958 ▸, 1959 ▸), which describes the concentration at each point of the simulated map as a function of time. After rescaling of parameters (Sappelt & Jäckle, 1997 ▸) and adding a temperature-jump dependency (Sciortino *et al.*, 1993 ▸) it can be written as 

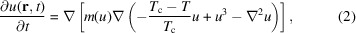

 where 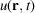 is rescaled time-dependent local concentration, *m*(*u*) is the mobility function and *T*_c_ is the critical temperature. The rescaled concentration can take values from −1 to 1. Positive values correspond to the dense phase, negative values correspond to the diluted phase. For classical spinodal decomposition, the mobility function *m*(*u*) is constant in time. Equation (2) was solved numerically on a 2D grid. Parameters of the simulation can be found in the supporting information.

The speckle pattern [*i.e.* the image in reciprocal space *I*(**q**, *t*_age_)] can be calculated for each time step as a square of the magnitude of the 2D fast Fourier transform of the fluctuations of the concentration (Barton *et al.*, 1998 ▸). The outcome is similar to the 2D scattering pattern obtained via XPCS–USAXS experiments. Following the same procedure as for the experimental data [equation (1)] we investigate the dynamics of the simulated LLPS process. Despite the quantitative difference between 2D and 3D simulations (Midya *et al.*, 2015 ▸; Desai & Kapral, 2009 ▸), the 2D CH simulations are sufficient for the qualitative analysis of the bulk dynamics of the system. The employed 2D CH model is considered to be a minimal model that captures the essence of the physical behavior (Toral *et al.*, 1995 ▸).

**Figure 2:**
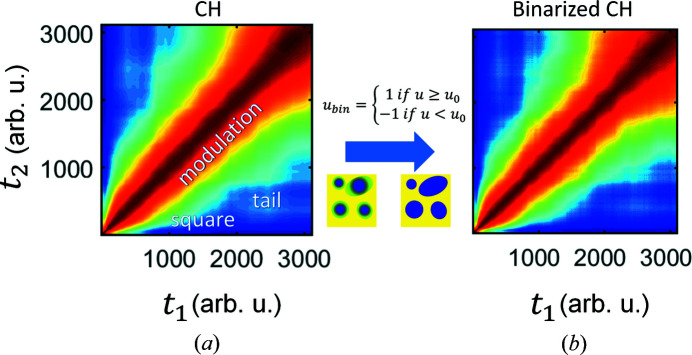
The result of the CH simulation is presented in (*a*) and represents similar features to the experiment (marked with text). (*b*) The TTC of the CH simulation with the binary modification of the 2D concentration field. Jet color bars of TTCs correspond to *G*(*q*, *t*_1_, *t*_2_) values. Above the blue arrow there is an equation used for binary modification and below there are schemes of the real-space 2D concentration field *u*(**r**, *t*) before and after binary modification. Parula color bars of the schemes correspond to the concentration distribution [from blue (−1) to yellow (1) color]. The time of CH simulations is in arbitrary units (arb. u.). The presented range of time in arb. u. of the CH simulation corresponds to the experimental time (see Fig. 5).

The main results of the simulations are presented in Fig. 2 ▸(*a*). As the resulting TTCs are *q* dependent, in order to qualitatively compare the simulation with experiment, we focus on a similar *q* region in comparison with the *q* value of the early-stage peak position of scattered intensity (Fig. S4). The total time of simulation was estimated based on the time *t*_age_-dependent scattering intensity at the chosen *q*. The simulated TTC reproduces the main features in the experimental TTC, *i.e.* modulation, square and tail. However, there are some obvious discrepancies between simulated and experimental TTCs. The square and tail features are significantly less pronounced in the simulation than in the experiment [compare Figs. 1 ▸ and 2 ▸(*a*)]. Furthermore, there is no change of the contrast along the diagonal of the simulated TTC. Importantly, the early stage of phase separation (∼30 s) is not reproduced by the simulation.

The typical strategy to overcome the discrepancies between the simulations and the experimental TTCs is to introduce a more sophisticated model with more parameters to optimize (*e.g.* CH with varying mobility, Navier–Stokes–Cahn–Hilliard). Unfortunately, this approach has many disadvantages. The CH equation-based models provide a simulated evolution of the real-space behavior. However, there are multiple sub-phenomena that can affect the resulting correlation maps: the emergence and disappearance of domains, their growth or dissolution, merging, changing the shape, variation of concentration at each point of the simulated pattern, *etc*. These sub-phenomena of LLPS may take place at the same time and depend on each other (*e.g.* evolution of concentrations and domain sizes). The only way we can investigate these processes is via changing the parameters of the model. However, the parameters affect the dynamics in a complex nonlinear way, which makes it challenging to study how different dynamical sub-phenomena influence TTCs independently.

To overcome this difficulty, we perform a direct modification in the simulated patterns. As the first step, we simplify the CH model via binarization of the simulated real-space patterns:

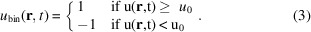

Surprisingly, despite the seemingly drastic changes, the resulting TTCs from a binary real space are virtually the same as from an initial CH simulation (Fig. 2 ▸). The only difference is the slightly less prominent square feature, which nevertheless appears at the same position. The fact that TTCs are nearly not affected by the binarization procedure is already a remarkable observation, and it inspires us to proceed with further simplifications of the model in order to study different aspects of the dynamics in an isolated and controllable fashion.

In this way, we can now consider a simplified simulation of the evolution of the binary domains and study how a TTC depends on different dynamical processes such as rates of growth and dissolution, domain sizes, *etc*. Thereby, in the following, we introduce a domain-based simulation with ‘manual’ control over all these dynamical processes of domains. Such an approach can be applied to various dynamical systems.

### 3.3. Reverse-engineering approach based on particle-based simulation

In order to simulate different phenomena such as growth, dissolution, merging, random-walk motion, shape/size/concentration distribution, as well as any combination of them with manual manipulation of all of the dynamical parameters, we model the simulated domains as spherical particles on a 2D map. Similar to the binarization approach described above, the concentration is constant inside the domains (*u*_dilute_ = −1 if not stated otherwise) and outside the domains (*u*_dense_ = 1). For each domain, we specify its radius and position as functions of time (see below). As a result of this heuristic approach, we obtain a 2D concentration map, representing our sample in real space, and its evolution with time. The corresponding TTC is calculated in the same way as discussed above for the CH simulation [equation (1)]. This will allow us to connect the various features in TTCs to the key control parameters of the phase-separation process.

#### 3.3.1. Modulation and tail – features of growth

**Figure 3:**
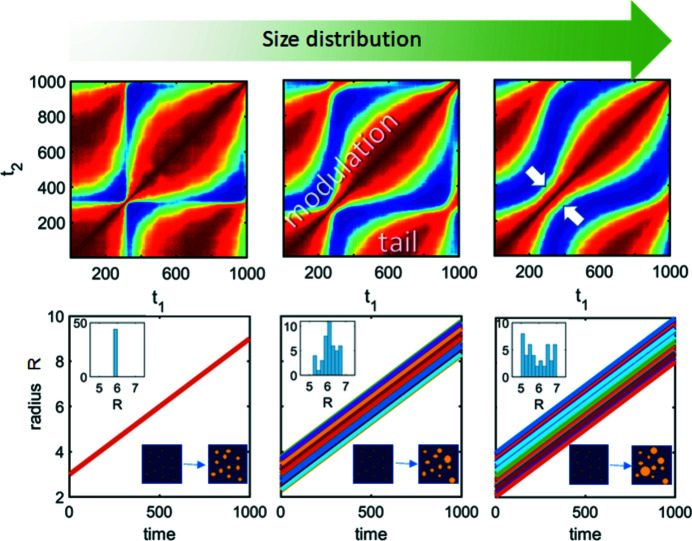
Particle-based simulation of linear growth with different radii distributions. The bottom row demonstrates the evolution of the radii of particles with time, with the real-space schemes in the right-bottom insets and the distribution of radii in the left-upper insets. Different colors correspond to different particles. The upper row of the figure shows the corresponding calculated TTCs at the same *q* value. White arrows point to the modulation waist in the TTC. The size distribution increases from the left to the right with the same behavior of the mean value.

To see how the domain growth influences the TTC, we simulated a set of spherical particles at random fixed positions and linearly growing radii with time (see Fig. 3 ▸). It is clear that there is a modulation of the relaxation time and the tail features appear in the TTCs. An increase in the size distribution results in a smearing of the modulation (the narrowest part of the modulations along the diagonal is marked with white arrows in Fig. 3 ▸).

The amplitude of the modulations depends on the initial and final sizes of the particles (Figs. 3 ▸ and S5); however, the final TTC also depends on their values at each point in time. The investigation of the nonlinear growth of particles (supporting information) demonstrates that their TTC is similar to the interpolation of the TTC of the linear growth corresponding to the evolution of the radii. Therefore, if the speed of growth is increasing with time, then the relaxation time is decreasing and vice versa. The uniformity of modulations depends on the speed of growth. The TTC is periodic only if the speed is constant, corresponding to the monotonic increase in the size of the particles. The larger the rate of the speed, the smaller the distance between adjacent modulation peaks. Thus, the TTC of the growth process calculated at a specific *q* value contains information about the evolution of the mean size and the distribution of particles of the whole system.

#### 3.3.2. Connecting domain size with the tail feature

**Figure 4:**
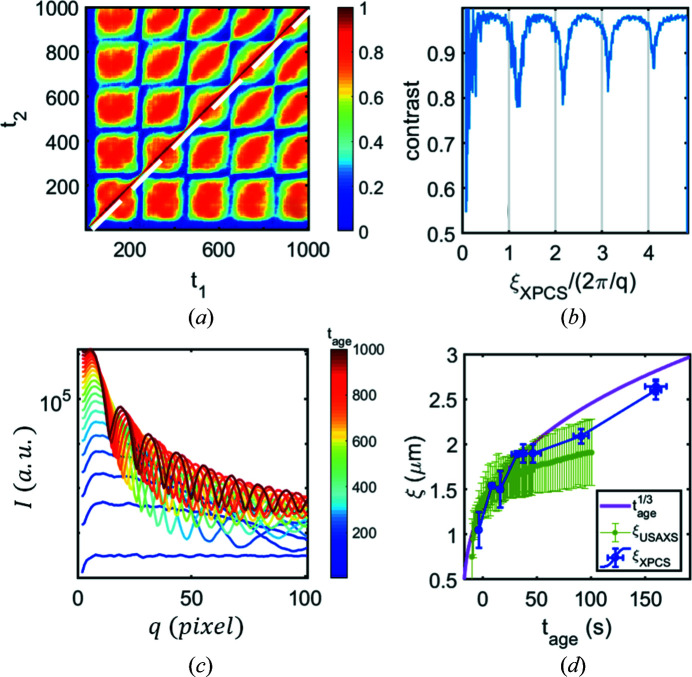
Extraction of the mean size for the particle-based simulation (*a*)–(*c*) and experiment (*d*). (*a*) TTC calculated for the model of identical particles, which grow with constant speed. (*b*) Dependency of contrast taken a pixel away from the diagonal in (*a*) (marked with a white dashed line) as a function of average size of the system ξ(*t*_age_) divided to the size 2π/*q* corresponding to the investigated *q* region. (*c*) Evolution of the intensity profile for the growth process. (*d*) Extraction of sizes from the experiment. The behavior of the characteristic size of the system, calculated with different methods. Characteristic length, obtained from the peak position in the scattering intensity ξ = 2π/*q*_peak_, is plotted for USAXS measurements. The purple line corresponds to the theoretically predicted increase of characteristic length with time following a power of 1/3 due to coarsening mechanisms based on diffusion or coalescence (Das *et al.*, 2015 ▸). Blue markers represent the average size of the domains calculated from XPCS measurements, following the appearance of tail features at the TTC for different *q* values.

We will further demonstrate here that the position/amount of tail features is connected with the mean size of domains. We assume that during the growth of domains, intensity peaks are shifting, and at a certain point for a given *q* value there can be a correlation between the *n*th peak at time *t*_age_ = *t*_*k*_ and the (*n* + 1)th peak at time *t*_age_ = *t*_*l*_, where *t*_*l*_ > *t*_*k*_ [Fig. 4 ▸(*c*)].

In our experiment, we observed the change in the evolution of intensity for fixed *q* due to a change of interparticle distance, which results in the shift of the peak of *I*(*q*) to smaller *q* values with time. Similar TTCs with tail features were recently obtained during growth on polycrystalline thin-film surfaces (Ju *et al.*, 2019 ▸), where multiple tail features were detected at the TTC and the oscillatory behavior of the *g*_2_ was observed. It was shown that the tail structure from Ju *et al.* (2019 ▸) appeared once the thickness of the deposited layer was equal to an integer multiple of the monolayer one.

Fig. 4 ▸(*b*) illustrates a similar effect for our simulations. The contrast profile [defined as a value of the TTC in Fig. 4 ▸(*a*) next to the diagonal] exhibits a periodic behavior as a function of time. It can be demonstrated that the period of the contrast is determined by the two key spatial parameters of the system. The first parameter is 2π/*q*, corresponding to the length scale where the dynamics were investigated. The second one is the time-dependent characteristic length ξ_XPCS_(*t*_age_), which for the simulation is the average particle diameter. By trivial recalculation of the contrast-profile time dependency into the characteristic length dependency, we can show that the tail appears approximately once the characteristic length of the system is an integer multiple of 2π/*q* [Fig. 4 ▸(*b*), Movie S2 of the supporting information]. Using this idea, experimental TTC maps with pronounced tail features were used for calculating ξ_XPCS_ to follow the microscopic growth kinetics of our BSA—YCl_3_ sample. The comparison with characteristic length, calculated from the peak position of the USAXS profiles ξ_USAXS_ = 2π/*q*_peak_, agrees well with ξ_XPCS_ obtained from the XPCS data [Fig. 4 ▸(*d*)]. According to theoretical predictions (Das *et al.*, 2015 ▸), the characteristic length should follow a power law 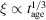 due to coarsening mechanisms based on diffusion or coalescence. However, it was found (Da Vela *et al.*, 2016 ▸) that the BSA—YCl_3_ system shows an arrested phase transition at high temperature, and the evolution of ξ cannot be described with classical spinodal decomposition theory. Therefore, the possibility to follow the time dependence of ξ is crucial for these types of systems (Da Vela *et al.*, 2016 ▸, 2017 ▸; Banc *et al.*, 2019 ▸). In Fig. 4 ▸(*d*) it is clearly seen that the ξ_USAXS_ plot is more complete; however, ξ_XPCS_ can be estimated from XPCS results even if the peak of the intensity profile (required for ξ_USAXS_ determination) moves out of the measurement window and a second order of *I*(*q*, *t*_age_) profile is not well pronounced. Such a possibility to extract sizes from the XPCS experiments may open new prospects for the experiments on growing, coarsening or evolving systems.

#### 3.3.3. Square feature caused by two-step processes

**Figure 5:**
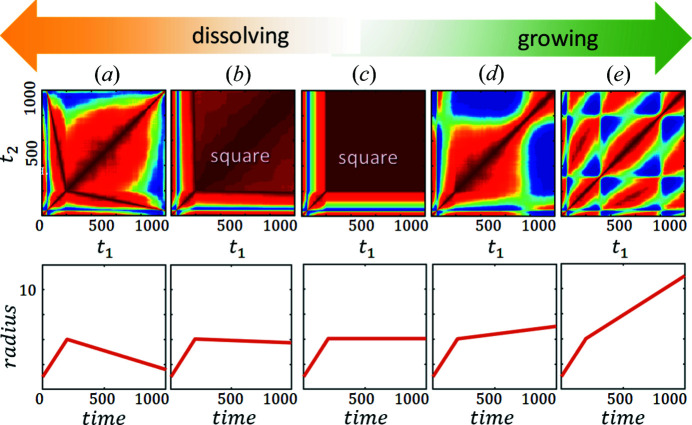
Particle-based simulation of a two-stage process. The bottom row demonstrates the evolution of the radii of particles with time. The upper row shows the corresponding calculated TTCs for the same *q* value. The speed of growth in the first stage (time < 200 arb. u.) is the same, followed by the different rates of the second stage. This stage is dissolution in (*a*) and (*b*), no change in size in (*c*), and growth in (*d*) and (*e*).

We have shown that the growth process results in the modulation and tail features on TTCs similar to the ones observed in the experiment. In previous works (Ragulskaya *et al.*, 2021 ▸; Girelli *et al.*, 2021 ▸), the correlation between the square feature and transition from the density-fluctuation stage to the coarsening stage of spinodal decomposition was shown. However, the subphenomena which impact the square feature have remained unclear. With the use of RE, we can show three possible ways of obtaining the square-like feature in the TTC (example in Figs. 5 ▸ and S6). The first case is a rapid change of the shape of the particles: time moments with similar particle shapes result in substantially higher correlation on the TTC compared with the moments with different particle shapes (Fig. S6). The second option is the rapid growth of particles followed by dissolution. As demonstrated in Fig. 5 ▸, the rate of growth should be much larger than the rate of dissolution. The third option is the rapid growth of contrast between the concentrations of particles and the background, so that the initial and final states have different standard deviations of the concentration distributions. The early stage of LLPS consists of the density fluctuation stage, and its transition to the coarsening stage is characterized by all of the options described above. The system undergoes a change of shape from worm-like structures to sphere-like domains of dilute phase surrounded by dense phase. Also, there is a rapid growth in the domain size (coupled with the change of the concentration), followed by dissolution of the smaller domains during an Ostwald ripening process (Voorhees, 1992 ▸) at the later coarsening stage. As was shown in Section 3.2, the concentration change is not the main process that impacts the square feature. Furthermore, the change of shape is not as drastic during the real LLPS as in the particle-based simulation. Thus, we will focus on the growth-dissolution behavior as the main process during LLPS.

The change in sizes of the domains during the LLPS can be simplified as a two-step process: a common growth in the early stage followed by the growth of one fraction of the particles and the dissolution of another (Ostwald ripening) in the coarsening stage. The particle-based simulation investigation of the impact on the TTCs from each of these components independently is presented in Fig. 5 ▸. For a better comparison, the first step, representing the growth of the particles, was the same for all processes. Furthermore, we assumed the same growth rate for all particles. If the second step is also growth [Figs. 5 ▸(*d*) and 5 ▸(*e*)], it results in the modulation and tail features on the TTC, as described in the previous section. The amount of these features depends on the speed in the second step [compare Fig. 5 ▸(*e*) with 5 ▸(*d*)]. If there is no change of size [Fig. 5 ▸(*c*)] or there is a slow dissolution in the second step, the square feature appears on the TTC [Fig. 5 ▸(*b*)]. The strength of this feature [value of *G*(*q*, *t*_1_, *t*_2_)] depends on the ratio between the rate of the growth and the rate of the subsequent dissolution. The slower the dissolution, the more visible the square feature [compare Fig. 5 ▸(*b*) with 5 ▸(*c*)]. Finally, if the rate of dissolution in the second step is similar to the rate of growth in the first step, the square feature completely disappears [Fig. 5 ▸(*a*)].

The square feature detected during the experiment is size sensitive: it appears earlier for larger *q* values [Fig. S2(*b*)]. This is consistent with the result of the particle-based simulation. Based on these findings, we suggest the following explanation of the square feature for USAXS–XPCS experiments on LLPS systems. During the Ostwald ripening process, small domains are the first to be dissolved. Since their size is inversely proportional to the value of *q*, these domains correspond to the high *q* values. Thus, the *q* dependency of the square feature is the result of the earlier transition from the growth stage to dissolution for small domains compared with the large ones.

#### 3.3.4. Brownian motion leads to reduction of feature visibility in TTCs

So far we have modeled the influence of kinetic parameters on the resulting TTCs. For these cases, 〈*I*(*q*)〉 (〈〉 – ensemble average) is not constant. The kinetics controlled features in TTCs are different from the speckle dynamics, which come from *I*(*q*) fluctuations with 〈*I*(*q*)〉 being constant. In the following, we introduce Brownian motion to the RE model, which is particularly important in the early stage of LLPS.

**Figure 6:**
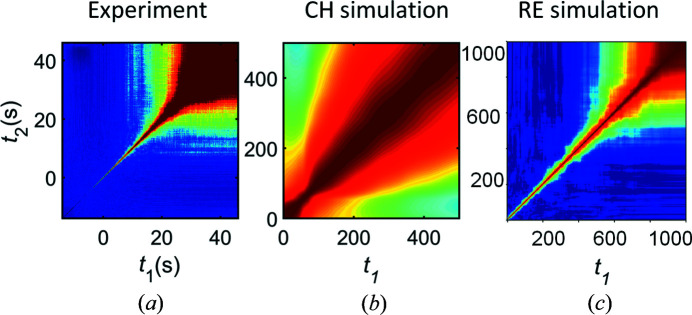
TTCs of the early stage of LLPS: for (*a*) experiment, (*b*) CH equation-based simulation and (*c*) the RE approach. (*a*) and (*b*) are initial parts of TTCs in Figs. 2 ▸(*a*) and 2 ▸(*b*), correspondingly, and (*c*) demonstrates the TTC of the particle-based simulation with the significant decrease in the rate of the Brownian motion with time and simultaneous growth of the particles (Fig. S9).

One of the main processes occurring at the early stage of LLPS is the growth of the domains. According to our particle-based simulation, we should expect modulations and a tail at the corresponding times on TTCs. These features appear in the CH simulation; however, the experimental behavior at the early stage of LLPS is different [Fig. 6 ▸(*a*)]: the relaxation time increases exponentially (Ragulskaya *et al.*, 2021 ▸; Girelli *et al.*, 2021 ▸), so the dynamics is very fast in the beginning and then quickly slows down; there are neither modulations nor a tail at the early stage. While it is well known that the early stage of the LLPS is sensitive to the thermal motion effects (Cook, 1970 ▸), our CH simulation does not include effects of thermal motion, a finite heating rate and viscoelastic properties of real systems, which may be the reason for the difference between simulation and experiment in the early stage of LLPS.

In our particle-based simulation, we model Brownian dynamics as a random-walk motion of the domains. The constant relaxation time on the TTC suggests equilibrium dynamics, which is consistent with the theory (Nogales & Fluerasu, 2016 ▸; Orsi *et al.*, 2012 ▸). An increase in the number of particles decreases the degree of heterogeneity. An increase in the rate of motion results in a decrease in the relaxation time (Fig. S7). The TTC varies from a constant *G*(*q*, *t*_1_, *t*_2_) = 1, when the Brownian motion is too slow, to a constant *G*(*q*, *t*_1_, *t*_2_) = 0, when the dynamics is too fast to be caught up within the simulated time scales (Fig. S7).

As a next step, the interplay between Brownian dynamics and growth is required. We fix the rate of the Brownian motion so that its effect is still visible in the TTC [Fig. S8(*a*)] and perform a set of particle-based simulations considering both, Brownian dynamics and growth [Figs. S8(*e*)–S8(*g*)], with the variation of the growth rate. It can be seen that if the growth is slower than the Brownian motion [compare Figs. S8(*b*) and S8(*c*) with S8(*a*)], the growth features are not seen in the TTC [Figs. S8(*e*) and S8(*f*)] and the Brownian motion effects are dominating. Once the rates of growth and Brownian motion become comparable, the characteristic features of growth (modulations and tail) become visible. Thus, the Brownian motion effects may be responsible for the reduction of the visibility of the growth features.

These results are consistent with our general intuition. When the Brownian motion is much slower than the growth rate, the domain positions remain essentially the same so TTCs look similar to the pure growth case. When the Brownian motion is much faster than the growth, the sizes of the particles for each moment remain essentially the same, resulting in TTCs similar to those of pure Brownian motion. When both of these rates can be obtained within the simulated (or experimental) time scale, the movement of the centers of the particles due to the Brownian motion effects results in the loss of the spatial correlations. This influences the visibility of the growth features and results in a correlation reduction of all the dynamical processes with the relaxation times higher than the characteristic time of the Brownian motion.

Incorporating Brownian motion into the particle-based simulation leads to TTCs exhibiting similar behavior to what we observe in the experiment [Figs. 6 ▸(*a*) and 6 ▸(*c*)]. This shows that the real system is highly influenced by the Brownian motion in the early stage. The rate of the Brownian motion is inversely proportional to the domain sizes, so the influence of Brownian motion on TTC features decreases with time due to the growth of the domains. Moreover, the viscosity of the dense phase increases, reducing Brownian motion even more. Therefore, the dynamics on the experimental TTC should be dominated by Brownian motion effects in the early stage with the gradual appearance of the features from other sub-phenomena of LLPS, and this effect becomes negligible in the coarsening stage. These arguments are in line with a previous investigation of the BSA–YCl_3_ system (Ragulskaya *et al.*, 2021 ▸). There, it was shown that the relaxation rate calculated from the mean intensity changes (reflecting the growth) is not visible on TTCs in the early stage and appears during the coarsening stage of LLPS. Thus, these results greatly expand our understanding of the dynamics we are measuring at the chosen length and time scales in the early stage of LLPS.

**Figure 7:**
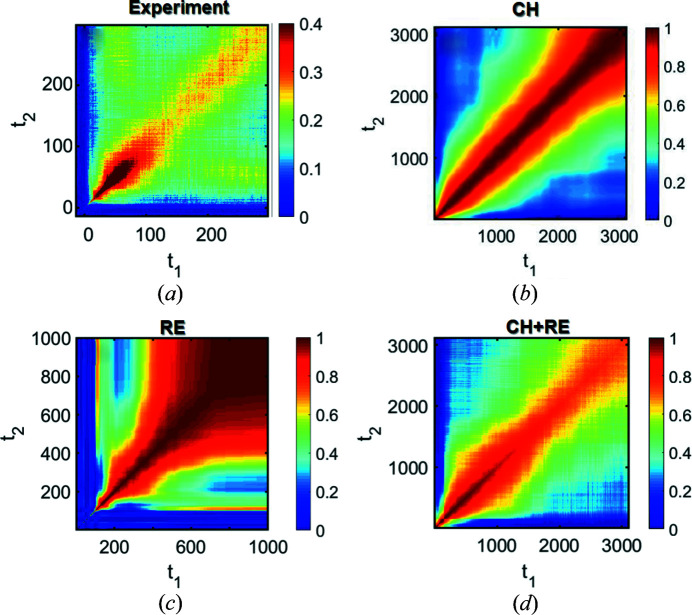
(*a*) Experimental TTC. (*b*) TTC obtained from the CH simulation. (*c*) An example of a TTC that is possible to obtain by RE with simple models (see Fig. S10). (*d*) A TTC obtained by modification of the evolution of the system by the CH equation with ideas (CH + RE) revealed from the RE. The ratio of speeds of growth in the early stage and dissolution in the coarsening stage was increased, which made the square feature more pronounced. Furthermore, the noise of 4% was added, which resulted in a change of the contrast along the diagonal similar to the experimental TTC.

In summary, in Section 3.3 we demonstrated that particle-based simulations allow one to successfully reverse-engineer the dynamics of the studied system and gain insights into nontrivial connections between the dynamical phenomena and the frequently observed TTC features. Using these it is possible to successfully reproduce the main features of the experimental TTC [compare Figs. 7 ▸(*a*) and 7 ▸(*c*)] by RE without the use of the equation-based theoretical models. This demonstration is helpful for the experiments that cannot be fully described by equation-based theoretical models, or if there is no theoretical formalism for the investigated phenomenon. Thus, the particle-based simulation itself is a significant addition to the conventional XPCS study. The results obtained here based on 2D simulations are conceptually consistent with 3D simulations (see Fig. S11), which are, of course, computationally much more expensive. In the next section, we connect this novel simulation-based RE with the established equation-based CH modeling of LLPS.

### 3.4. Application of reverse engineering to Cahn–Hilliard simulations

In this section, we extend the RE approach and demonstrate the possibility to combine it with regular theoretical equation-based simulations by modifying the simulated real space accordingly for a better understanding of the studied system. In the following, we apply some very basic changes to overcome the discrepancies between the experiment and the classical CH simulations based on the RE analysis. As mentioned earlier, in comparison with the experiment, CH simulations exhibit a different early stage, pronunciation of the square feature and no contrast variation along the diagonal. The first issue, as demonstrated above, is related to thermal motion. Here, we discuss the other two problems.

The RE method showed that the brightness of the square feature is mostly dependent on the value of the ratio between the growth rate of the early stage and the dissolution rate in the coarsening stage. This idea can be checked now with the classical CH simulation. As a first step, a binary modification of the 2D concentration field was performed to simplify the later modifications and interpretations [Figs. 2 ▸ and S12(*b*)]. Later, the change in growth- to dissolution-rates ratio was modified by adding additional pixels at each step of the simulation to the calculated boundaries of the domains (Fig. S12). It can be seen that the bigger this ratio of rates, the more pronounced is the square feature (Fig. S13).

The increase in the ratio of the speed of growth in the early stage to the speed of dissolution in the coarsening stage in comparison with the classical CH is a signature for the gel/glass transition. It shows that the dissolution of the particles during the Ostwald ripening process is slower than it was supposed to be in the classical system with constant mobility. Thus, for a more precise theory-based simulation of the chosen experimental system, the classical CH should be replaced with the CH with the change of the mobility corresponding to the gelation process (Sappelt & Jäckle, 1997 ▸). Having a closer look for such types of simulations [*e.g.* (Girelli *et al.*, 2021 ▸)], one may notice that the closer a system is to the gel state, the more pronounced the square feature for both experiment and simulation.

Another remarkable difference of CH from the experimental TTC is that there are no fluctuations in the contrast along the diagonal. We found that adding randomly distributed low-intensity noise with a constant amplitude to the scattering pattern of the CH simulations results in the same behavior of contrast as for the experiment (see the supporting information for details). The impact of the noise level on the classical CH simulated maps is presented in Fig. S14. The change in the contrast is accompanied by a change in the scattering intensity [Fig. S4(*b*)]; so with the constant level of noise, there is a change in the signal-to-noise ratio, resulting in contrast fluctuations. According to the simulation, around 4% from *I*_max_ of noise is required to mimic the experimental results at the investigated *q* value. This value was compared with the experimentally measured data to validate the process.

Finally, as a demonstration of Section 3.4, we present the CH simulation modified by RE and noise as a scattering pattern [Fig. 7 ▸(*d*)]. This modification of CH by RE is closer to the experimental TTC than pure CH simulation [compare Figs. 7 ▸(*d*) and 7 ▸(*b*) with 7 ▸(*a*)]. Thus, the modification provides a better understanding of the investigated system.

## 4. Conclusions

In this work, we proposed an RE approach to predict and understand the kinetics and dynamics of systems undergoing non-equilibrium processes. As an example, we studied the LLPS in a BSA–YCl_3_ system by XPCS in the USAXS mode. TTCs for the investigated *q* values show three characteristic features: modulation, square and tail. Based on the RE approach, we revealed the following connections. Firstly, the modulation, which is usually the main component for studying dynamics with XPCS, corresponds to the growth of the domains, where the growth rate decreases with time (increasing the relaxation time).

We also found that rapid change of shape and concentration distribution and also a change from the rapid growth of domains at the early stage to subsequent slow dissolution result in the square-like feature of the TTC. All of these sub-phenomena are characteristic of the LLPS systems going from the density fluctuation stage to the coarsening stage of spinodal decomposition. However, the RE-modified CH simulations revealed that in the real system the ratio of growth rate in the early stage to the speed of dissolution in the coarsening stage during Ostwald ripening has the largest impact on the square feature. We showed that this rate should be higher than predicted by the classical CH theory, which is a signature of the gelation process.

The tail feature arises from correlation of the system between different distant times and has a strong *q* dependence, appearing earlier for higher *q* values. This phenomenon may be caused by the shift of scattering intensity *I*(*q*) due to change in size and mean interparticle distance as a result of the correlation between different harmonics, and it appears once the characteristic length of the domains is a multiple of the investigation box size (2π/*q*). Based on this result, we calculated the characteristic length of the experimental system from XPCS and showed that it follows the temporal evolution of the characteristic length calculated from USAXS, which demonstrates that microscopic dynamics and kinetics are intimately intertwined.

Furthermore, we demonstrated that the fluctuation of the contrast along the diagonal is mainly a result of the incoherent low-intensity noise rather than a feature of the LLPS process.

These results demonstrate that the observed side features in TTCs are not experimental artefacts but rather a rich source of dynamic information of the system. The RE approach can go beyond CH theory and build the connection between these features in the TTC and the key parameters, such as relaxation time, concentration distribution, the size distribution of the domains, viscosity and mobility. The RE approach also shows the importance of taking into account thermal motion effects in the early stage of the LLPS. Finally, the framework established in this work can be employed for various types of systems and processes such as growth, coarsening or evolving systems, and many other phenomena that are essential for understanding the fundamentals of materials synthesis, processing and phase transformation. One of the primary benefits of this algorithm is the ability to go beyond the existing theory if the experiment cannot be fully described by it or if there is no theoretical formalism for the investigated phenomenon.

## 5. Conflicts of interest

There are no conflicts to declare.

## 6. Related literature

The following references are cited in the supporting information for this article: Guo *et al.* (2010) ▸, Madsen *et al.* (2018) ▸, Robinson *et al.* (2003) ▸. 

## Supplementary Material

Supporting information including experimental details, simulation parameters and more advanced RE simulation. DOI: 10.1107/S2052252522004560/ti5024sup1.pdf

Movie S1. Evolution of speckle distribution. DOI: 10.1107/S2052252522004560/ti5024sup2.avi

Movie S2. The q-dependency of the two-time correlation function calculated for the model of identical particles, which grow with constant speed (left), and corresponding contrast evolution (right). The tail feature appears once the characteristic length of the domains is a multiple of the investigation box size. DOI: 10.1107/S2052252522004560/ti5024sup3.wmv
