# Supplementary material for: Reverse-engineering method for XPCS studies of non-equilibrium dynamics
Source: IUCrJ. 2022 May 28;9(Pt 4):439–48. doi: 10.1107/S2052252522004560 (PMC9252156; doi:10.1107/S2052252522004560)
Supplement: Supplementary file 1 [file m-09-00439-sup1.pdf]

IUCrJ

Volume 9 (2022)

Supporting information for article:

Reverse-engineering method for XPCS studies of non-equilibrium dynamics

Anastasia Ragulskaya, Vladimir Starostin, Nafisa Begam, Anita Girelli, Hendrik Rahmann, Mario Reiser, Fabian Westermeier, Michael Sprung, Fajun Zhang, Christian Gutt and Frank Schreiber

Supporting information. Reverse-engineering method for XPCS studies of non-equilibrium dynamics.

Anastasia Ragulskaya,[a*] Vladimir Starostin,[a] Nafisa Begam,[a] Anita Girelli,[a] Hendrik Rahmann,[b] Mario Reiser,[b,c] Fabian Westermeier,[d] Michael Sprung,[d] Fajun Zhang,[a] Christian Gutt[b] and Frank Schreiber[a]

[a] *Institute of Applied Physics, University of Tübingen, Auf der Morgenstelle 10, 72076, Tübingen, Germany,* [b] *Department of Physics, University of Siegen, Emmy-Noether-Campus, Walter-Flex-Str. 3, 57076, Siegen, Germany,* [c] *European X-ray free-electron laser GmbH, Holzkoppel 4, 22869, Schenefeld, Germany, and* [d] *Deutsches Elektronen-Synchrotron DESY, Notkestraße 85, D-22607 Hamburg, Germany. E-mail: anastasia.ragulskaya@uni-tuebingen.de*

XPCS; **LLPS**; **Proteins**; **Reverse-Engineering**; **Cahn-Hilliard**

1. Experimental parameters.

XPCS experiments were performed in the USAXS mode at the PETRA III beamline P10 (DESY, Hamburg, Germany) at an incident X-ray energy of 8.54 keV ($\lambda = 1.452$ Å) and beam size of 100 $\mu m \times 100$ μm. The sample to detector distance was 21.2 m, which corresponds to the q range of 3.2×10^{-3} nm^{-1} to 3.35×10^{-2} nm^{-1}, where $q = 4\pi/\lambda \cdot \sin\theta$ and 2θ is the scattering angle. Taking signal-to-noise ratio and beamstop into account, the q range for the XPCS data analysis is from 3.5 to 11.2 μm^{-1}. The

data were collected by an EIGER X 4M detector from Dectris with 75 μm × 75 μm pixel size. The sample was filled into capillaries of 1.5 mm in diameter and first equilibrated at 10 °C for 10 minutes. Then, it was heated by the Linkam heating stage to 40 °C with a heating rate of 150 °C/min and measured for 312 s and 60 s from the beginning of the heating. After the measurement, the solution was cooled back to 10 °C. Each measurement was taken on a fresh sample spot separated by larger than the beam size distance from the previous one to avoid the beam damage due to the long exposures. The number of frames, exposure time, and delay time (SI Table 1) were calculated based on the beam damage test, which was done preliminary to all measurements. The sample was measured with different attenuators at 10 °C, so that we checked the behavior of the sample under the beam in the one-phase regime. The time of survival of the sample was considered as time with no change in USAXS profile and simultaneous noise in TTC and equaled to 60 s under the flux 1.42×10^9 ph/s, which was taken into account for all further measurements. The temperature of the sample was recorded every 2 seconds. First 60 s and 312 s experiments were done under different exposure parameters (while under the beam damage threshold). However, the overlapping time period demonstrated the same kinetics and dynamics, which is additional evidence for selecting the beam parameters that do not affect the sample.

2. Parameters of the CH simulation

Equation 2 (main text) was solved numerically on a 2D grid 256×256 for 34500 time steps with $\Delta t = 0.09$ (SI Fig. 3 (c)-(f)). The initial concentration for each point in space was defined as random noise fluctuations with amplitude 0.05 around $u_0 = 0.4$, which corresponds to the dense phase (similar to the experiment) so that these noise fluctuations have a uniform distribution. The temperature T was set to $0.05T_c$. The

resulting TTC is the average over three simulations.

3. Nonlinear growth

In the main text it was shown that the growth process results in the "tail" features in the TTC (section 3.3.1). Results for the linear growth are presented in Fig. 3 of the main text. It was shown that the amplitude of the modulations depends on the initial and final sizes of the particles (Fig. 3 and SI Fig. 5), however, the final TTC also depends on their values at each point in time. This can be seen comparison of the particle-based simulation of linear and nonlinear growth. SI Fig. 5 (a) and (c) show TTCs of the particle system with linear (b) and exponential (d) growth of radii, respectively, with the same initial and final values. Both of the TTCs demonstrate 1.5 modulations, however, the shape of the "tail" feature and the period of modulation depends on the function of the rate of growth. The exponential growth with different boundary conditions (SI Fig. 5 (c) and (e)) results in the same shapes of TTC features, distinct from linear growth (SI Fig. 5 (a)). This result can be easily understood (follow the "star" in SI Fig. 5); for instance, a linearly growing system reaches $radius = 6$ at $time \approx 500$ (b), while exponentially growing systems - at $time \approx 800$ (d) and ≈ 350 (f), respectively. These time points result in exactly the same environment in the TTCs: they are located on the first half of the second modulation feature (as indicated in the SI Fig. 5 (a), (c) and (e) by the yellow star). A similar procedure can be performed with any values of the size parameter *radius*. Finally, one can find that the TTC in (c) is similar to the non-linear interpolation of the TTC in (a) corresponding to the non-linear behavior of the radii of particles with time.

4. Low-intensity noise as a reason for the contrast variation in TTC.

Another remarkable difference of CH from the experimental TTC is that there are no fluctuations in the contrast along the diagonal. One of the typical explanations of such behavior is the existence of additional fast motions. their contributions change with time and that is beyond the time resolution of the XPCS measurement. The following data procedure demands the extraction of the localization length (Guo *et al.*, 2010; Madsen *et al.*, 2018). However, it is not expected on the time-scale of the measurement for this sample and does not appear in the CH simulation. So, there should be another explanation. The behavior of the contrast is reflected in the evolution of the distribution of speckles (SI Movie S1). We found that adding randomly distributed low-intensity noise with a constant amplitude to the scattering pattern of the CH simulations results in the same behavior of contrast as for the experiment. The impact of the noise level on the classical CH simulated maps is presented in SI Fig. 14. The change in the contrast is accompanied by a change in the scattering intensity (SI Fig. 4 (b)), so with the constant level of noise, there is a change in the signal-to-noise ratio, resulting in contrast fluctuations. According to the simulation around 4% from I_{max} of noise is required to mimic the experimental results at the investigated q-value. This value was compared to the experimentally measured data to validate the process. The most prominent source of noise in the experiment is the scattering background by all incoherently scattering objects (air, water), which change so fast that they are completely incoherent in the time range of a single image acquisition (Robinson *et al.*, 2003). Its value can be estimated by the initial scattering of the sample before the LLPS started and is in line with the simulation results.

5. Table and figures

Table 1. *Experimental parameters*

Duration (s)	Number of frames	Exposure (s)	Delay (s)	∼ Time of average (s)
60	7500	0.008	0	0.08
312	3000	0.004	0.1	1

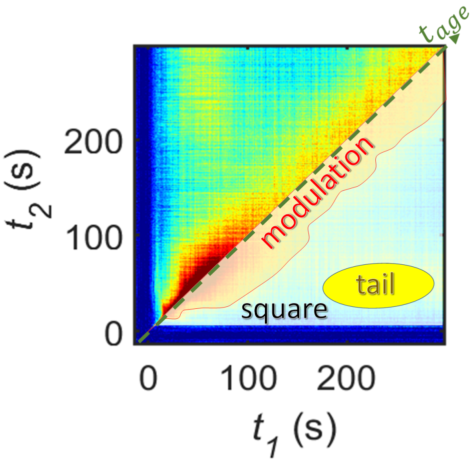

Fig. 1. Experimental two-time correlation map presented in Fig. 1 of the main text. Shaded patches mark the regions of features of the TTC. White shade for "square", red for "modulation" and yellow for "tail". The green dashed line shows a direction of increase of experimental time t_{age}.

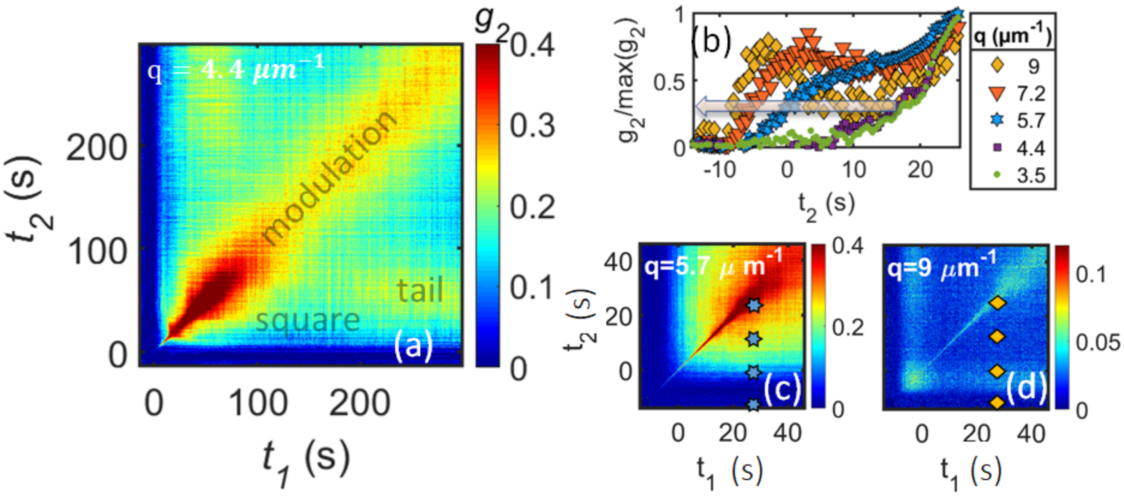

Fig. 2. Two-time correlation functions for temperature jump from 10 °C to 40 °C for 312 s (a) and for 60 s experiments (c-d) for different q-values. Color bars correspond to $G(q, t_1, t_2)$ values. On (a) features of the TTC ("modulation", "square" and "tail") are displayed with a half-transparent grey text. (b) "Square" feature dependency can be caught by the normalized vertical cuts of TTC at $t_2 = 30s$ (follow blue stars on (c) and orange diamonds on (d)) for different q. A rapid increase in the values corresponds to the beginning of the "square" feature at the TTC. It is shifting to earlier t_2 with an increase of q (grey arrow).

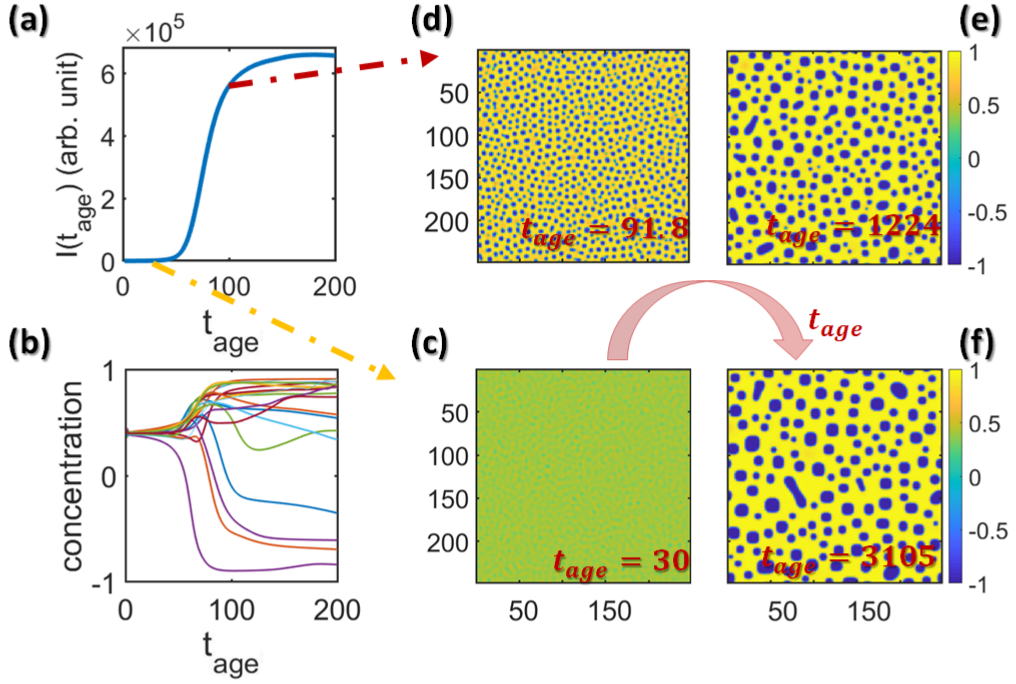

Fig. 3. (a) Behavior of scattered intensity with t_{age} for the early stage of LLPS (t_{age} <200) for $T = 0.05T_c$ for simulated data for the same $q = 22pixel$ for which Fig. 3 in the manuscript was presented. (b) Change of concentration for different random points (one point - one line) in the real space with time t_{age} during the early stage of LLPS. Time of significant change in concentration functions has a similar value to the time of jump of structure factor on (a). (c)- (f) Simulated real space pictures of the process of LLPS for different t_{age} in the 248×248 pixels square. Colormap represents normalized concentration u(m,n, t_{age}) values. Yellow and Red dash-dot lines show the correspondence of the structure factor value and state of the system in the real space at the same time t_{age}.

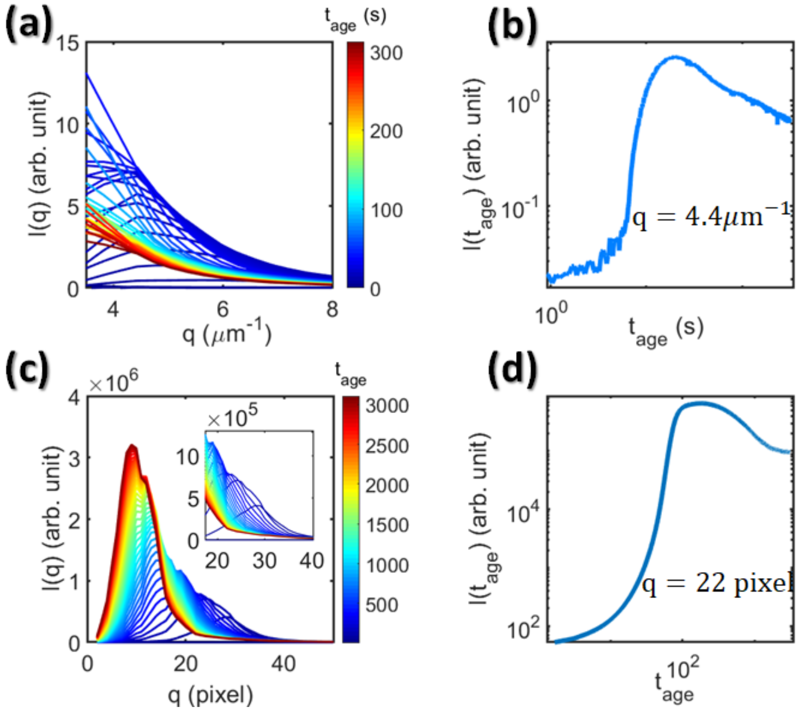

Fig. 4. (a) Intensity profiles obtained by USAXS experiment at different experimental times t_{age} for T= 40°C. (b) Intensity at $q = 4.4\mu m^{-1}$ and T= 40°C. (c) Simulated intensity profiles obtained from equation 2 for $T = 0.05T_c$. In the right corner there is a zoom for $q = [20, 40]$ pixel range. (d) Intensity for simulated data for $T = 0.05T_c$ at $q = 22$ pixels. It is clearly seen that $q = 22$ pixels of the simulated intensity profile and $q = 4.4\mu m^{-1}$ of the experimental profile have the same behavior (compare (d) with (b)) and these q–values are similar relative to the peak position of scattered intensity in the beginning (compare picture in the right corner of (c) with (a)).

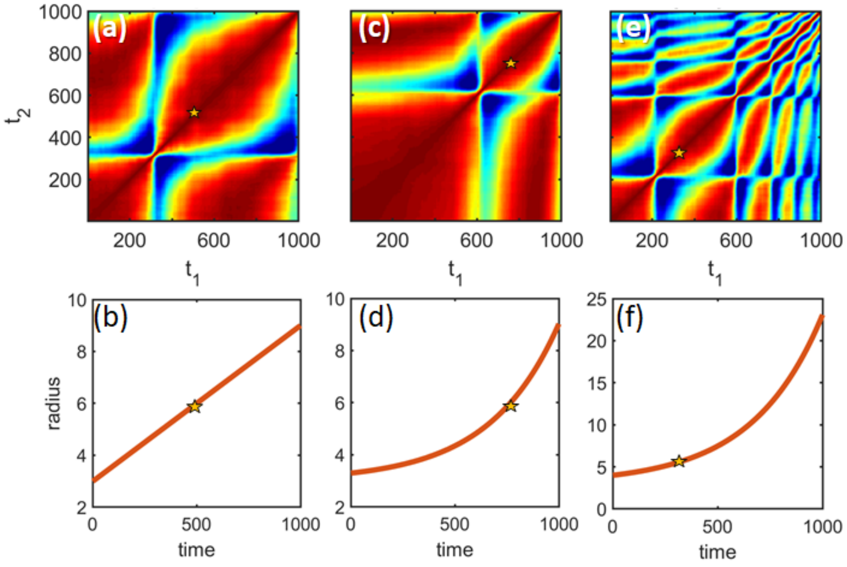

Fig. 5. Particle-based simulation of growth with different speed. The bottom row demonstrates the evolution of the radii of particles with time. The upper row shows the corresponding calculated TTCs for the same q-value. (a-b) shows linear growth of particles from $radii = 3$ to 9 pixels.(c-d) represents exponential growth of particles from $radii =$3 to 9 pixels. (e-f) shows the exponential growth of particles from $radii =$3 to 23 pixels. The yellow star with a black border shows when the $radii = 6$ for different simulations. These time points result in exactly the same position on a modulation feature while the features are transformed according to the non-linear behavior of the radii of particles with time.

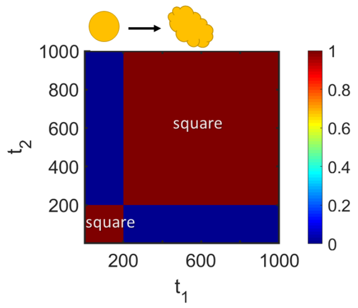

Fig. 6. Particle-based simulation of the rapid change of shape. Moments with similar shape of particles correlate much better than with different ones: $t_1, t_2 = [0, 200]$ and $t_1, t_2 = [200, 1000]$.

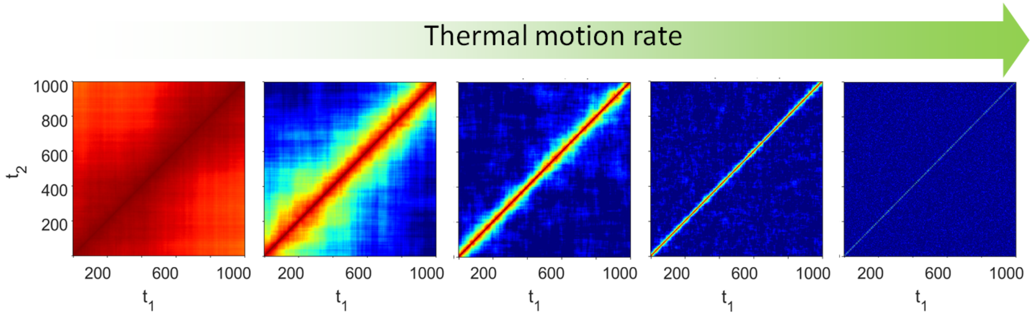

Fig. 7. Particle-based simulation of the Brownian motion effects. The rate of dynamics increases from left to right. Faster is the Brownian dynamics, smaller is the relaxation time in the TTC.

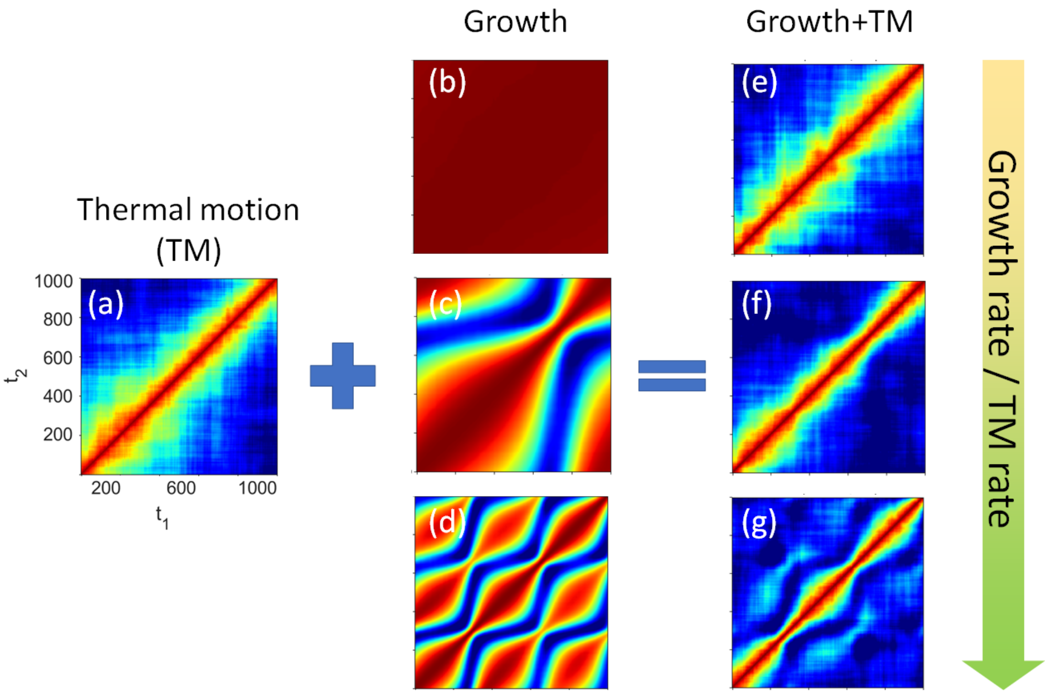

Fig. 8. Particle-based simulation of the interplay between growth and Brownian motion effects. The TTC of the pure Brownian motion with the constant rate (a). The TTC of the pure growth (b-d) with different rates, increasing from (b) to (d). The simulation (e-g) of the simultaneous growth (with parameters from (b-d)) and Brownian motion (with parameters from (a)). The ratio between growth rate and Brownian motion rate is increasing from (e) to (g). The Brownian motion effects on TTC dominate if the growth is slower (e, f).

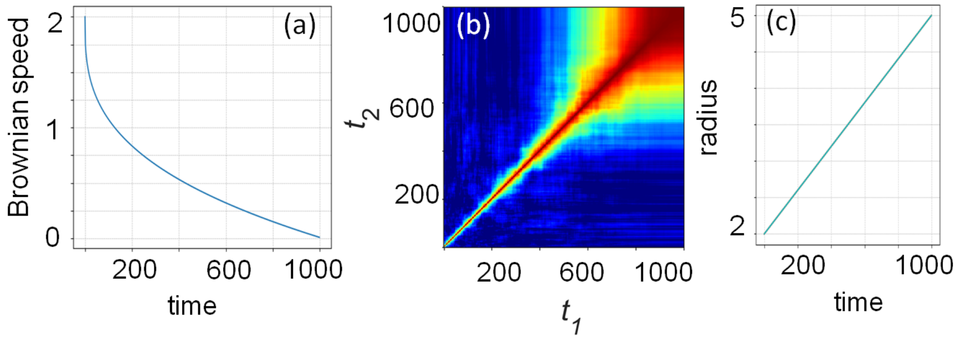

Fig. 9. Particle-based simulation of the Brownian motion with the change in the Brownian motion rate with time and simultaneous growth. (a) Evolution of the Brownian motion rate. (c) Evolution of the radii of particles. (b) Corresponding TTC (also presented in Fig. 6 (c) in the main text).

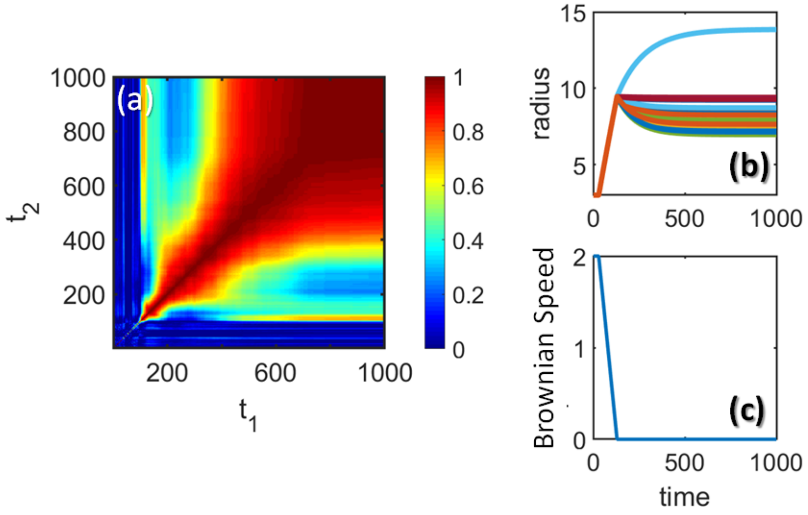

Fig. 10. Example of TTC, which is possible to obtain by RE with simple models (a). Corresponding evolution of sizes of the particles (b) and Brownian motion speed (c).

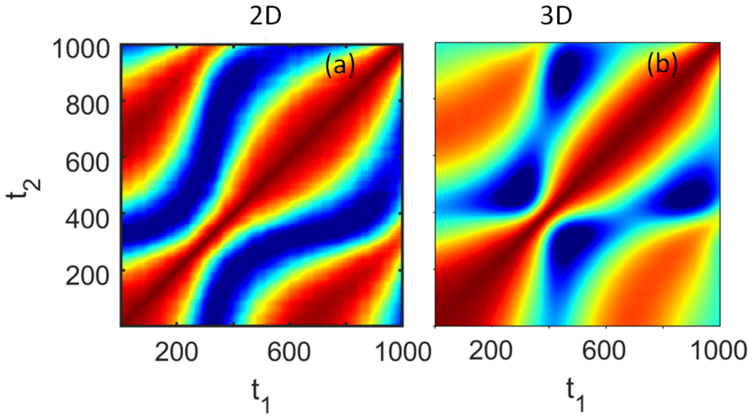

Fig. 11. Particle-based simulation of linear growth in 2D (a) (presented in Fig. 3 in the main text) and 3D (b) exhibiting similar features.

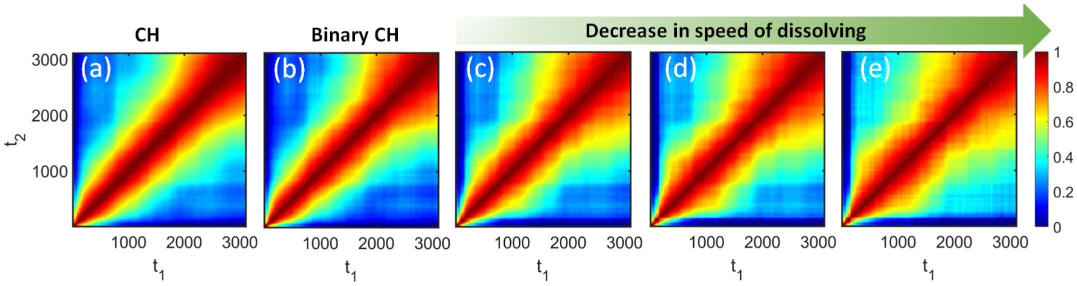

Fig. 12. Two-time correlation maps obtained by modification of CH simulation with the particle-based simulation. (a) Classical CH (same to Fig. 2 (a) in the main text). (b) The binary modification of 2D concentration field. (c-e) Modification of the binary CH by manual increasing of the domain boundaries resulted in the decrease in speed of dissolution. The speed of dissolution is decreasing from (c) to (e).

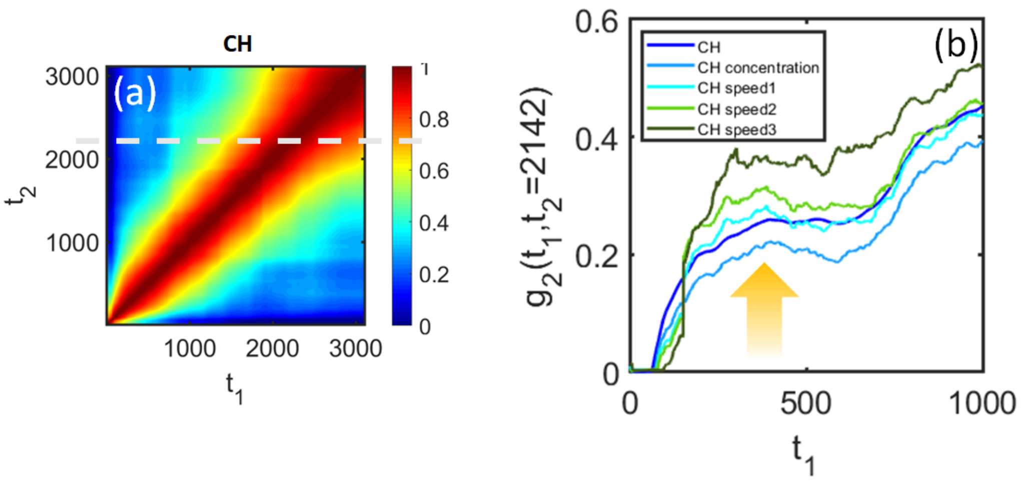

Fig. 13. Investigation of the impact of different parameters on the "square" feature pronunciation. For each set of the parameters TTC was calculated, then the cut at $t_2 = 2142$ was made to quantify the "square" feature (**a**). The "square" feature results in the raise jump in the $g_2(t_1, t = 2142)$ curves (pointed by arrow on (**b**)). The bigger is this jump, the more pronounced the "square" feature is. The absence of the evolution (e.g. putting domain concentration as -1 and background concentration as 1 for all times) is labeled as "CH concentration" on (**b**) and results in a minor change of the "square" pronunciation (compare "CH concentration" curve with the results of the classical Cahn-Hilliard "CH"). Other curves correspond to the manual modification of the velocity ratio of growth in the early stage and dissolution in the coarsening stage with the absence of the concentration dependency for the real-space maps calculated by the classical CH. The higher is the number on labels ("CH speed 1", "CH speed 2","CH speed 3"), the higher was the ratio introduced. It is clearly seen that the increase of this ratio results in the more pronounced "square" feature (follow "CH concentration" with no change in ratio in comparison to the classical CH, "CH speed 1", "CH speed 2" and "CH speed 3").

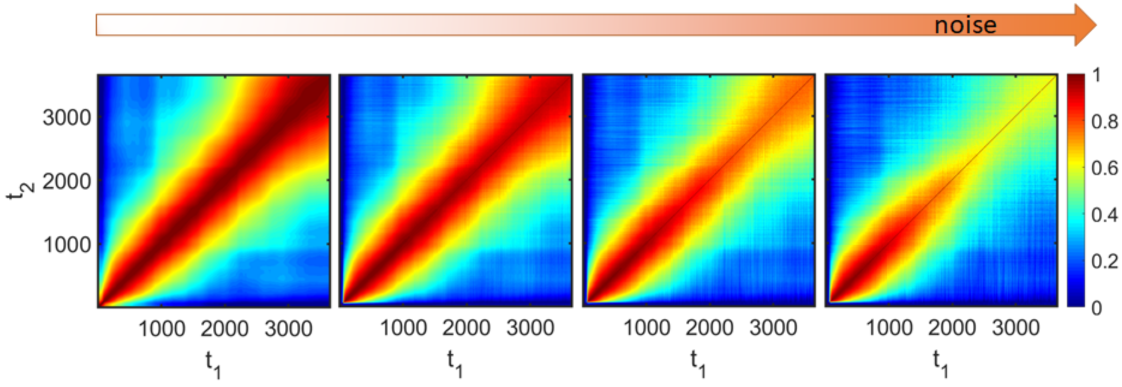

Fig. 14. The impact of the noise level on the classical CH simulated TTC maps. The randomly distributed noise was added to the scattering intensity calculated by the Fourier Transform of the real space simulated image. The maximum noise level amplitude $(max(I_{noise})/max(I) * 100\%)$ varies from 0% on the left till around 8% on the right. The most prominent noise source in the experiment is the scattering background by all incoherently scattering objects (air, water), which change so fast that they are completely incoherent in the time range of single image acquisition.

References

Guo, H., Ramakrishnan, S., Harden, J. L. & Leheny, R. L. (2010). *Phys. Rev. E*, **81**, 050401. https://link.aps.org/doi/10.1103/PhysRevE.81.050401

Madsen, A., Fluerasu, A. & Ruta, B. (2018). *Structural Dynamics of Materials Probed by X-Ray Photon Correlation Spectroscopy*, pp. 1–30. Cham: Springer International Publishing. https://doi.org/10.1007/978-3-319-04507-8_29-2

Robinson, I., Kenney-Benson, C. & Vartanyants, I. (2003). *Physica B: Condensed Matter*, **336**(1), 56 – 62. Proceedings of the Seventh International Conference on Surface X-ray and Neutron Scattering. http://www.sciencedirect.com/science/article/pii/S0921452603002692
